# Supplementary material for: Global forest fragmentation change from 2000 to 2020
Source: Nat Commun. 2023 Jul 11;14:3752. doi: 10.1038/s41467-023-39221-x (PMC10336092; doi:10.1038/s41467-023-39221-x)
Supplement: Supplementary file 1 — Supplementary Information [file 41467_2023_39221_MOESM1_ESM.pdf]

Supplementary Information for

Global forest fragmentation change from 2000 to 2020

Jun Ma[#, *], Jiawei Li[#], Wanben Wu & Jiajia Liu*

Ministry of Education Key Laboratory for Biodiversity Science and Ecological Engineering, Coastal Ecosystems Research Station of the Yangtze River Estuary, Institute of Biodiversty Science, School of Life Sciences, Fudan University, #2005 Songhu Road, Shanghai, 200438, China.

# These authors contributed equally to this work.

* Corresponding author:
Jun Ma, ma_jun@fudan.edu.cn
Jiajia Liu, liujiajia@fudan.edu.cn

The Supplementary Information contains Supplementary Figures 1-8 and Supplementary Note 1.

Supplementary Figures

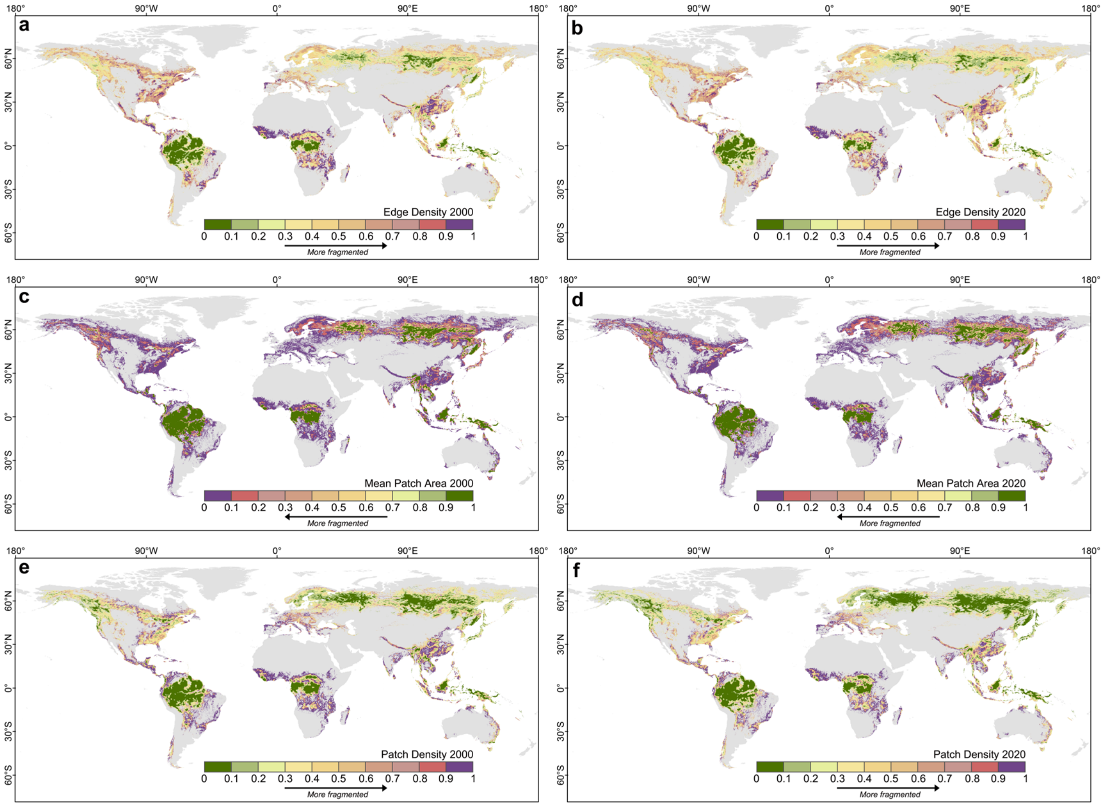

Supplementary Fig. 1 | The spatial distribution of **(a-b)** edge density (ED), **(c-d)** mean patch area (MPA), and **(e-f)** patch density (PD) for global forest landscapes in 2000 and 2020. The range of values for each landscape pattern index is 0-1. More details of the specific calculation formulas were mentioned in the Methods section of the main text and Extended methods section of the Supplementary Information.

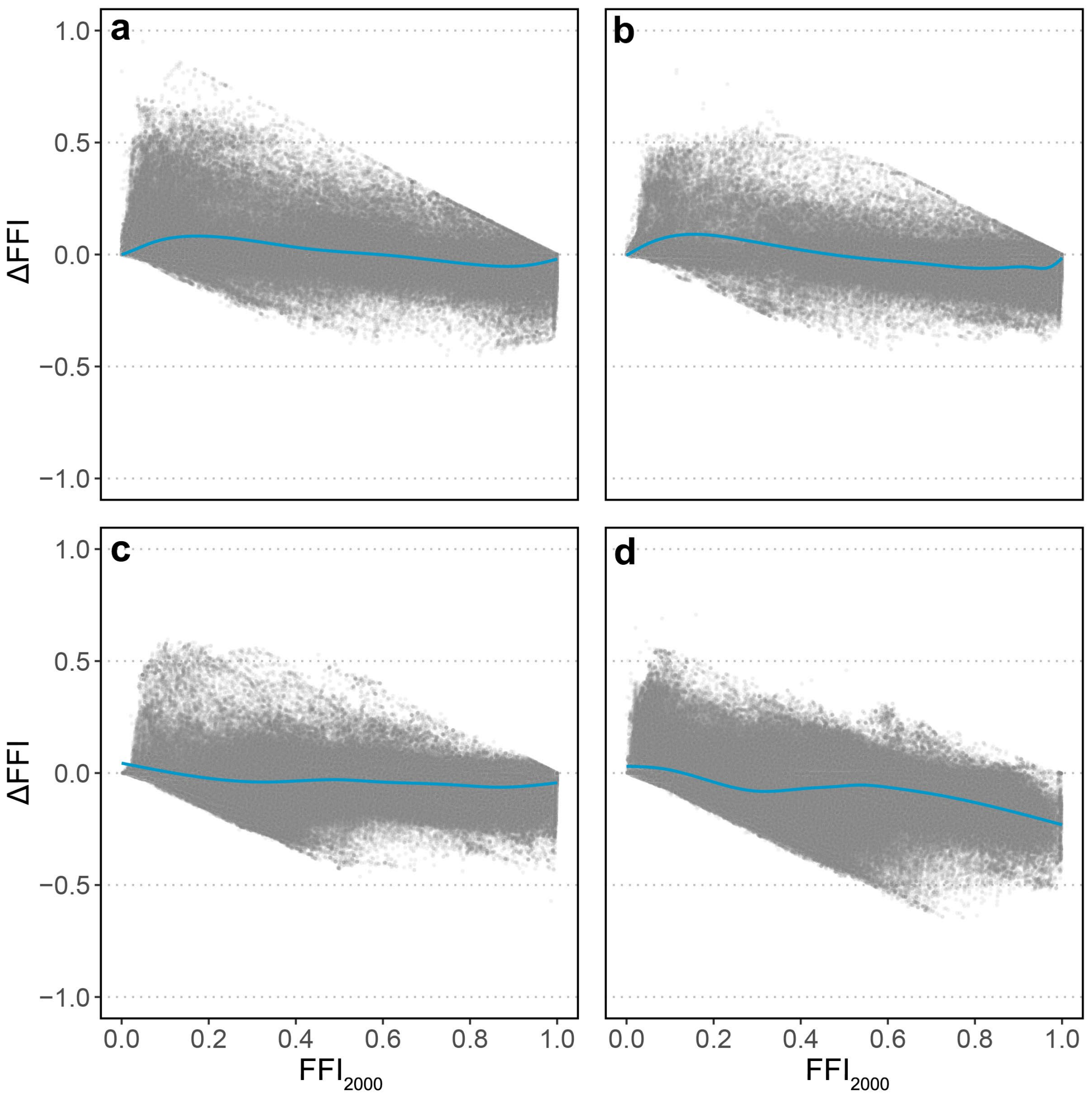

Supplementary Fig. 2 | The relationships between the static forest fragmentation index (FFI_{2000}) and the dynamic forest fragmentation index during 2000-2020 (ΔFFI) for **(a)** Tropical zone, **(b)** Subtropical zone, **(c)** Temperate zone, and **(d)** Boreal zone. The blue curve was plotted using the generalized additive model (GAM) method. The darker the color of the dots indicated the more dots in this region.

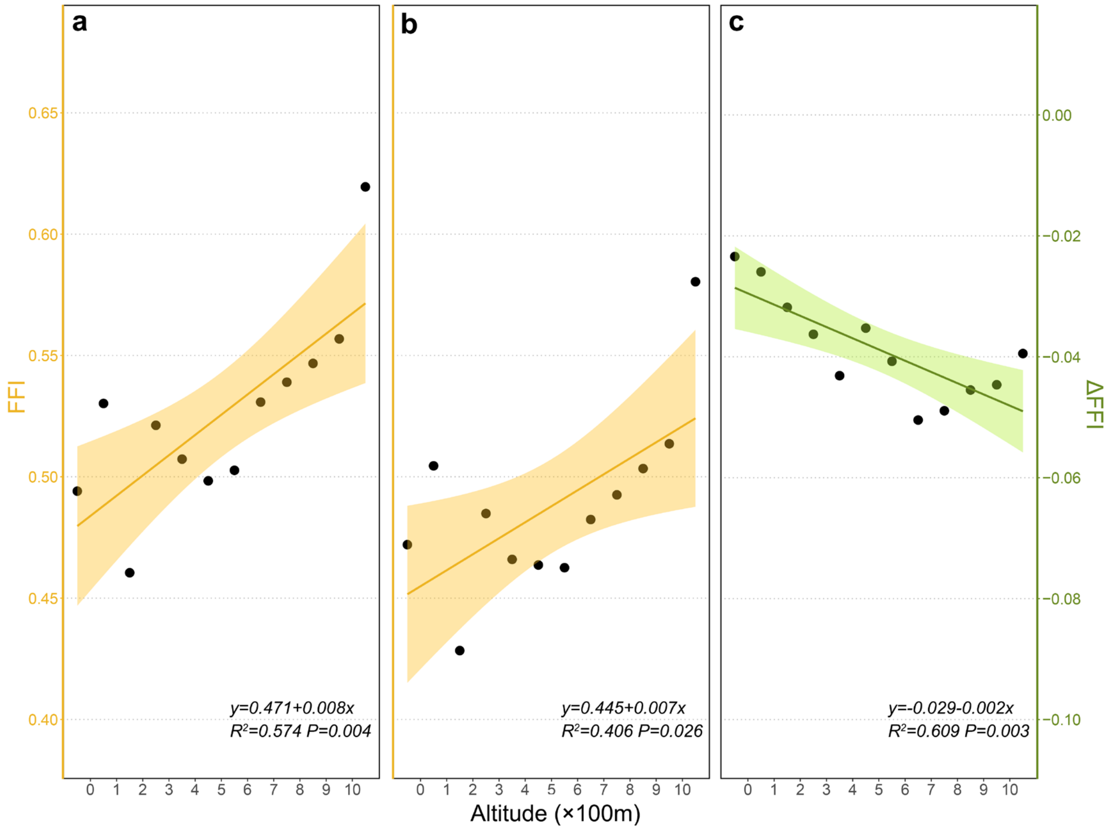

Supplementary Fig. 3 | The relationships between **(a-b)** the static forest fragmentation indexes (FFI_{2000} and FFI_{2020}; orange) and **(c)** the dynamic forest fragmentation index during 2000-2020 (ΔFFI; green) with altitude gradient. The black dots indicated the mean value of FFIs and ΔFFI in each 100m altitude interval. The dark-colored lines indicated the one-dimensional linear regression curve and the light-colored error bands indicated the 95% confidence intervals of the fitted models based on these black dots. The corresponding y-axis labels were the same color as the fitted curve. The statistical significance in **(a-c)** was obtained with a two-side Student's T-test and the relevant parameters of the models were indicated.

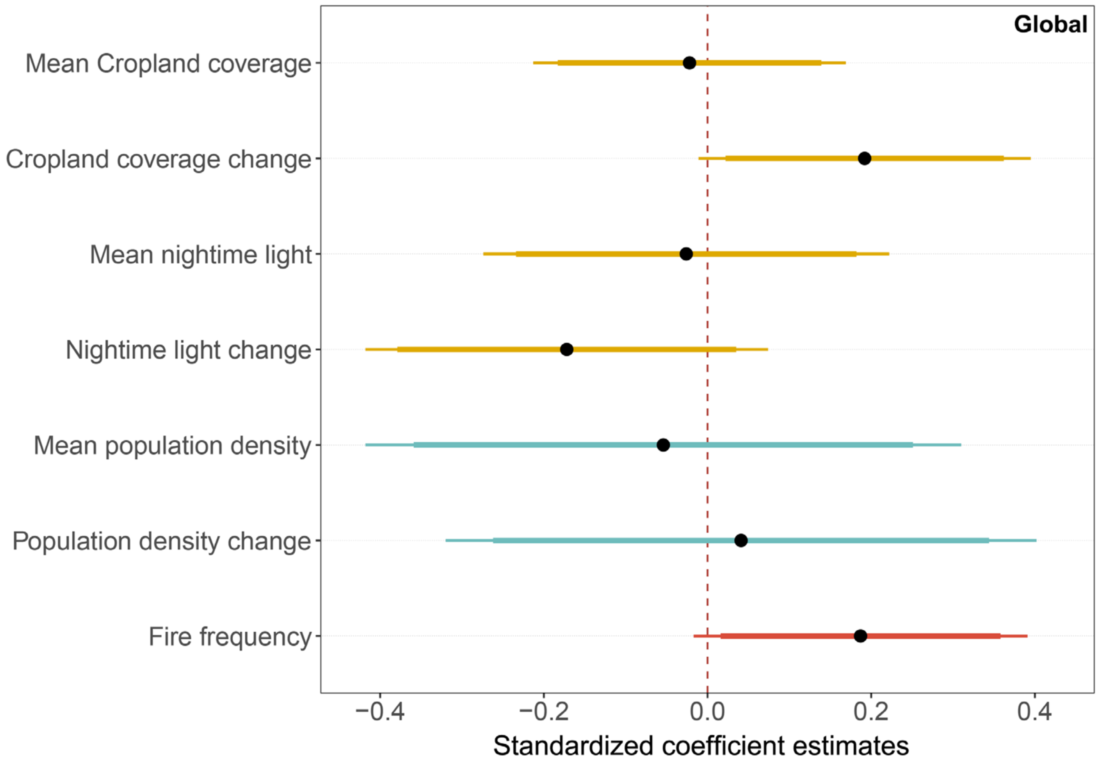

Supplementary Fig. 4 | Standardized correlation coefficients of the dynamic forest fragmentation index (ΔFFI) for the globe. Relative effects of anthropogenic activity (the mean and difference values of cropland coverage and nighttime light; yellow color), demographic pressure (the mean and difference values of population density; blue color), and natural disturbance (fire frequency; red color) on dynamics of ΔFFI of all forested countries (n=131 countries). The dots represented standardized coefficient estimates with 95% (thin segments, ± 1.960 standard errors) and 90% (thick segments, ± 1.645 standard errors) confidence intervals in generalized linear models.

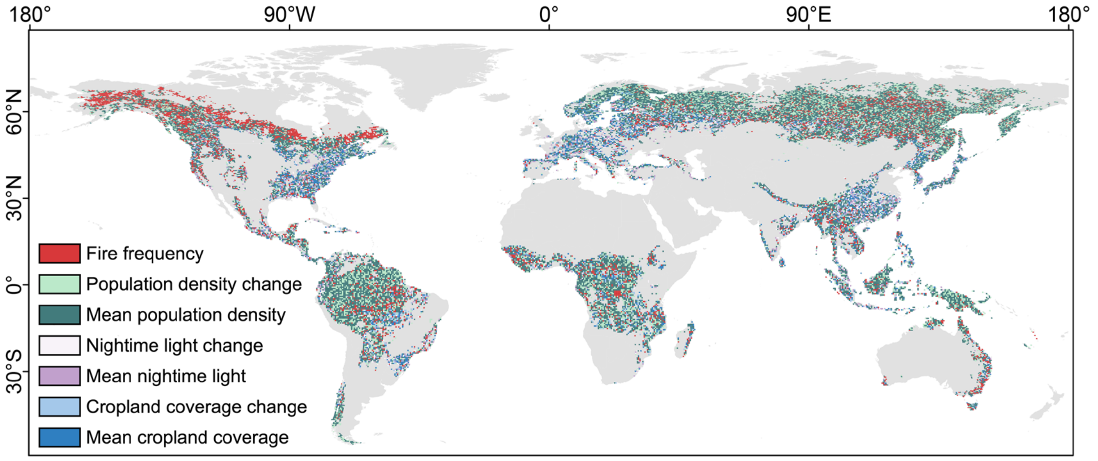

Supplementary Fig. 5 | Primary drivers of the dynamic forest fragmentation index (ΔFFI) for the period 2000 to 2020. The primary driver factor for each 50 km grid was represented by the factor with the highest absolute value of standardized coefficient estimates.

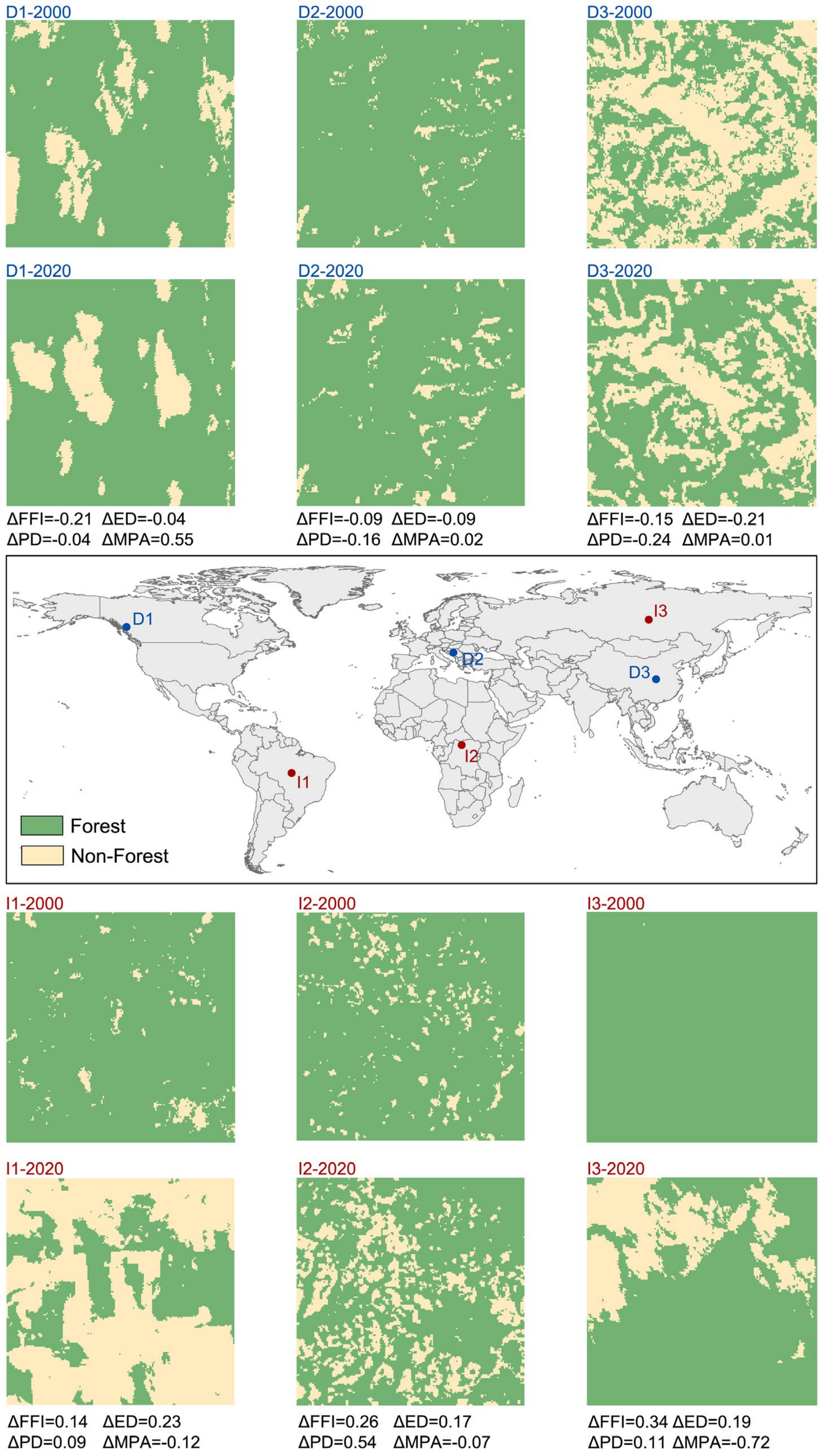

Supplementary Fig. 6 | Spatial distribution of full resolution of forest cover change from 2000 to 2020 for some sites of different locations of the globe. Each of the site is a 5 km size grid and was selected in relevant hotspots in forest fragmentation decreased and increased areas of **Fig. 2**.

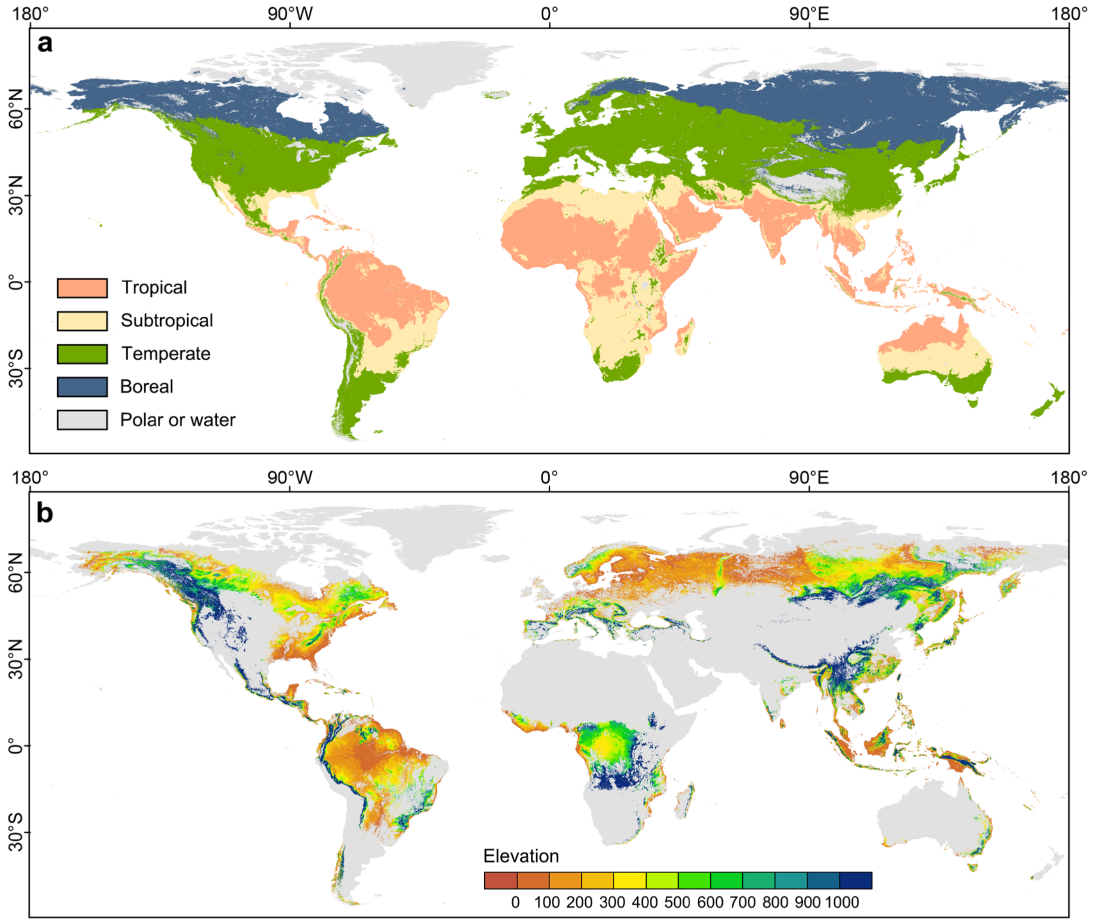

Supplementary Fig. 7 | (a) Global climate zones map and **(b)** elevation map for forest area. The sources of these data were described in detail in the Methods section of the main text.

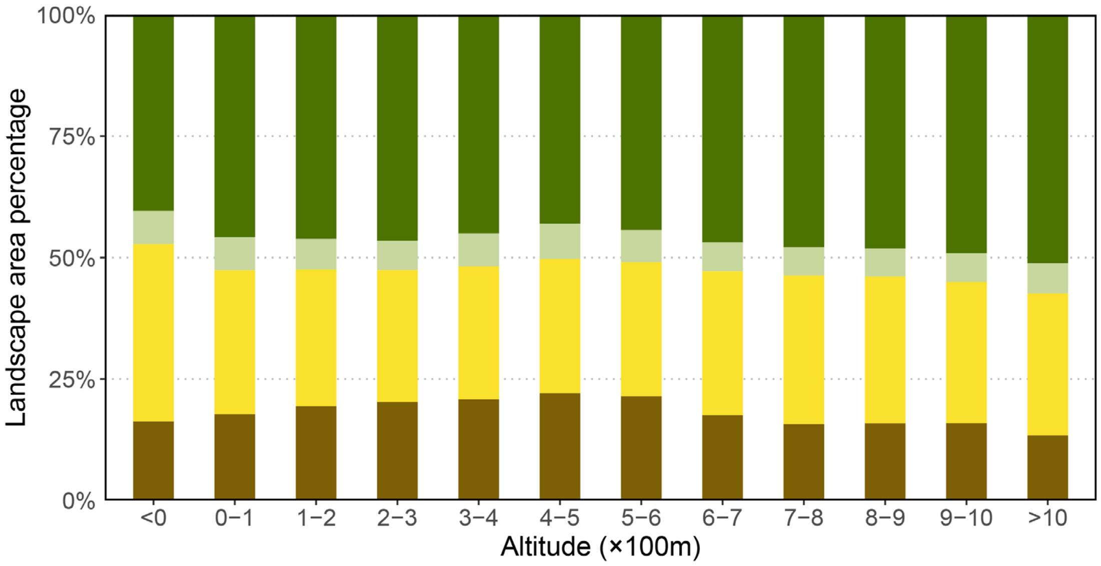

Supplementary Fig. 8 | The area percentage of four-forest landscape dynamic patterns in altitudinal gradient. The brown bars indicated $FC_{down}FFI^{up}$ ($\Delta FC < 0$ and $\Delta FFI > 0$), the yellow bars indicated $FC_{down}FFI_{down}$ ($\Delta FC < 0$ and $\Delta FFI < 0$), the light green bars indicated $FC^{up}FFI^{up}$ ($\Delta FC > 0$ and $\Delta FFI > 0$) and the dark green bars indicated $FC^{up}FFI_{down}$ ($\Delta FC > 0$ and $\Delta FFI < 0$). The specific classification was described in detail in the Methods section of the main text.

Supplementary Note 1

The specific processes for the calculation of the static forest fragmentation index

We constructed a synthesized forest fragmentation index (FFI) using the normalized single fragmentation metrics. During the normalization of ED, PD, and MPA, both the directions of the three metrics in reflecting forest fragmentation and the comparability of the FFI in different years were considered. The upper and lower limit values of the landscape pattern metrics in 2020 were first obtained to identify outliers based on the formula as follows:

$$Q_{upper} = Q_3 + 1.5\mathrm{IQR} \quad (1)$$

$$Q_{lower} = Q_1 - 1.5\mathrm{IQR} \quad (2)$$

where Q_{upper} and Q_{lower} represent the upper and lower limit values for each landscape pattern metric, respectively; Q_1 is the first quartile (lower quartile); Q_3 is the third quartile (upper quartile) of each landscape pattern metric after equal division into quartiles; and IQR denotes the quartile distance, i.e., the difference between Q_3 and Q_1. This method can represent the distribution of the data more realistically and intuitively because landscape pattern metrics did not obey the normal distribution. We set the pixel values that were beyond the upper and lower limits as the corresponding limit values to exclude the influence of extreme outliers of the data.

Then, taking the upper and lower limit values established for 2020 as the standard, the values for 2000 were processed similarly so that the data from the two years could be compared horizontally. The normalization was carried out using the following equation:

$$Y_{nor} = \frac{X - X_{min}}{X_{max} - X_{min}} \quad (3)$$

where Y_{nor} represents the normalized results for ED, PD and MPA with a range of 0-1, X is the original value of ED, PD or MPA, and X_{max} and X_{min} are the corresponding maximum and minimum values, respectively.

Finally, the three landscape pattern metrics were jointly used to construct the static forest fragmentation index (FFI) with average weight:

$$FFI_k = \frac{ED_{nor} + PD_{nor} + (1 - MPA_{nor})}{3} \quad (4)$$

where k is the specific year and FFI_k is the static forest fragmentation index with a range of 0-1. We then defined three levels of global forest fragmentation: low (0-0.2), medium (0.2-0.8) and high (0.8-1).
