# Supplementary material for: Global forest fragmentation change from 2000 to 2020
Source: Nat Commun. 2023 Jul 11;14:3752. doi: 10.1038/s41467-023-39221-x (PMC10336092; doi:10.1038/s41467-023-39221-x)
Supplement: Supplementary file 3 — Description of additional supplementary files [file 41467_2023_39221_MOESM3_ESM.pdf]

<u>Description of additional supplementary files</u>

Supplementary Data 1**:** Mean values of the changes in forest coverage (ΔFC) during 2000-2020, the dynamic forest fragmentation index (ΔFFI) during 2000-2020, the static forest fragmentation indexes (FFI_{2000} and FFI_{2020}) in 131 countries around the world.
