# Supplementary material for: Global forest fragmentation change from 2000 to 2020
Source: Nat Commun. 2023 Jul 11;14:3752. doi: 10.1038/s41467-023-39221-x (PMC10336092; doi:10.1038/s41467-023-39221-x)
Supplement: Supplementary file 5 — Reporting Summary [file 41467_2023_39221_MOESM5_ESM.pdf]

nature portfolio

Corresponding author(s): Jun Ma and Jiajia Liu

Last updated by author(s): May 20, 2023

Reporting Summary

Nature Portfolio wishes to improve the reproducibility of the work that we publish. This form provides structure for consistency and transparency in reporting. For further information on Nature Portfolio policies, see our Editorial Policies and the Editorial Policy Checklist.

Statistics

For all statistical analyses, confirm that the following items are present in the figure legend, table legend, main text, or Methods section.

n/a	Confirmed	
[ ]	[x]	The exact sample size (*n*) for each experimental group/condition, given as a discrete number and unit of measurement
[x]	[ ]	A statement on whether measurements were taken from distinct samples or whether the same sample was measured repeatedly
[ ]	[x]	The statistical test(s) used AND whether they are one- or two-sided<br>*Only common tests should be described solely by name; describe more complex techniques in the Methods section.*
[x]	[ ]	A description of all covariates tested
[ ]	[x]	A description of any assumptions or corrections, such as tests of normality and adjustment for multiple comparisons
[ ]	[x]	A full description of the statistical parameters including central tendency (e.g. means) or other basic estimates (e.g. regression coefficient) AND variation (e.g. standard deviation) or associated estimates of uncertainty (e.g. confidence intervals)
[ ]	[x]	For null hypothesis testing, the test statistic (e.g. *F*, *t*, *r*) with confidence intervals, effect sizes, degrees of freedom and *P* value noted<br>*Give P values as exact values whenever suitable.*
[x]	[ ]	For Bayesian analysis, information on the choice of priors and Markov chain Monte Carlo settings
[x]	[ ]	For hierarchical and complex designs, identification of the appropriate level for tests and full reporting of outcomes
[x]	[ ]	Estimates of effect sizes (e.g. Cohen's *d*, Pearson's *r*), indicating how they were calculated

Our web collection on statistics for biologists contains articles on many of the points above.

Software and code

Policy information about availability of computer code

Data collection	No software was used.
Data analysis	Data analysis was performed using R version 4.2.2 and ArcGIS version 10.8. We used the 'landscapemetrics' package (version 2.0.0) in R to calculate three landscape pattern indices, and then normalized the three indices using the maximum-minimum method. Finally, we obtained the forest fragmentation index (FFI) using the average-weight method. More details of the data and code we used have been described in previous studies, and we have annotated the manuscript with relevant references.

For manuscripts utilizing custom algorithms or software that are central to the research but not yet described in published literature, software must be made available to editors and reviewers. We strongly encourage code deposition in a community repository (e.g. GitHub). See the Nature Portfolio guidelines for submitting code & software for further information.

Data

Policy information about availability of data

All manuscripts must include a data availability statement. This statement should provide the following information, where applicable:

- Accession codes, unique identifiers, or web links for publicly available datasets
- A description of any restrictions on data availability
- For clinical datasets or third party data, please ensure that the statement adheres to our policy

All data used in the analysis are publicly accessible. The global land cover and land use change dataset is available at https://glad.umd.edu/dataset/GLCLUC2020 (including forest cover data and cropland coverage data); The global climate zones dataset is available at https://storymaps.arcgis.com/

stories/61a5d4e9494f46c2b520a984b2398f3b; The global altitude dataset is available at https://ngdc.noaa.gov/mgg/topo/gltiles.html; The global NPP-VIIRS-like nighttime light dataset is available at https://dataverse.harvard.edu/dataset.xhtml?persistentId=doi:10.7910/DVN/YGIVCD; The worldPop global gridded population count dataset is available at https://hub.worldpop.org/project/categories?id=3; The MODIS monthly global burned area dataset is available at https://lpdaac.usgs.gov/products/mcd64a1v006/. The global administrative boundary dataset is available at https://data.apps.fao.org/map/catalog/static/search?format=shapefile. The Forest Fragmentation Index (FFI) data generated in this study have been deposited in the Figshare repository at https://figshare.com/s/21dbf1f50250aeb7f5a0.

Human research participants

Policy information about studies involving human research participants and Sex and Gender in Research.

Reporting on sex and gender	Not applicable.
Population characteristics	Not applicable.
Recruitment	Not applicable.
Ethics oversight	Not applicable.

Note that full information on the approval of the study protocol must also be provided in the manuscript.

Field-specific reporting

Please select the one below that is the best fit for your research. If you are not sure, read the appropriate sections before making your selection.

☐ Life sciences ☐ Behavioural & social sciences ☒ Ecological, evolutionary & environmental sciences

For a reference copy of the document with all sections, see nature.com/documents/nr-reporting-summary-flat.pdf

Ecological, evolutionary & environmental sciences study design

All studies must disclose on these points even when the disclosure is negative.

Study description	This study mapped global distribution of forest fragments and their temporal changes between 2000 and 2020 based on remote sensing data. The results showed that 75% of the world's forests experienced a decrease in fragmentation, while tropical forests experienced the most severe fragmentation over the past two decades.
Research sample	We used global forest cover data provided by satellite, which can be available at https://glad.umd.edu/dataset/GLCLUC2020. All forest pixels in this dataset were our research samples. Based on these forest pixels, we analyzed the fragmentation of global forests according to their spatial patterns.
Sampling strategy	We used satellite remote sensing data to map global forest fragmentation. We calculated multiple landscape pattern indices from the class scale within each 5 km window range and obtained the static forest fragmentation index (FFI) for each window using the weighted average method to characterize the degree of global forest fragmentation.
Data collection	All datasets were downloaded directly from Internet using the URLs in the data availability statement. The forest cover data and cropland coverage data were produced by Potapov, P. et al.; the altitude data was produced by GLOBE Task Team; the nighttime light data was produced by Chen, Z. et al.; and fire data sourced from MCD64A1 version 6.These data were all obtained from a series of different satellites. The climatic zone data was produced by Sayre, R. et al.; the population data was produced by Lloyd, C. T. et al. and the administrative boundary data was produced by the Food and Agriculture Organization (FAO). These data were produced by combining remote sensing data and survey data.
Timing and spatial scale	The basic data for the study, the static forest fragmentation index (FFI), is remote sensing data with a spatial resolution of 5 km in 2000 and 2000
Data exclusions	We excluded pixels with forest coverage below 30% under a 5 km window to determine the global forest extent.
Reproducibility	Our analyses were based on global high-resolution remote sensing data, and we have provided data source information in the data availability statement. Based on the source data we used and the FFI data we produced, which have been deposited in the Figshare repository, all of our research results can be reproduced by referring to the methods section.
Randomization	Randomization was not relevant to our study. Because our study was based on satellite remote sensing data to map and analyze global forest fragmentation, and does not require similar experimental content.
Blinding	Blinding was not relevant to our study. Because our study was based on satellite remote sensing data, it does not involve sample collection or selection.

Did the study involve field work? ☐ Yes ☒ No

Reporting for specific materials, systems and methods

We require information from authors about some types of materials, experimental systems and methods used in many studies. Here, indicate whether each material, system or method listed is relevant to your study. If you are not sure if a list item applies to your research, read the appropriate section before selecting a response.

Materials & experimental systems

n/a	Involved in the study
☒	☐ Antibodies
☒	☐ Eukaryotic cell lines
☒	☐ Palaeontology and archaeology
☒	☐ Animals and other organisms
☒	☐ Clinical data
☒	☐ Dual use research of concern

Methods

n/a	Involved in the study
☒	☐ ChIP-seq
☒	☐ Flow cytometry
☒	☐ MRI-based neuroimaging
